# Continual reassessment method for dose escalation clinical trials in oncology: a comparison of prior skeleton approaches using AZD3514 data

**DOI:** 10.1186/s12885-016-2702-6

**Published:** 2016-08-31

**Authors:** Gareth D. James, Stefan N. Symeonides, Jayne Marshall, Julia Young, Glen Clack

**Affiliations:** 1 PHASTAR, Unit 2, 2a Bollo Lane, London, W4 5LE UK; 2 AstraZeneca, Alderley Park, Macclesfield, Cheshire SK10 4TF UK; 3 Edinburgh Cancer Research Centre, University of Edinburgh, Western General Hospital, Edinburgh, EH4 2XR UK

**Keywords:** Clinical trial, Phase 1, Continual reassessment method, Skeleton, Bayesian, Oncology

## Abstract

**Background:**

The continual reassessment method (CRM) requires an underlying model of the dose-toxicity relationship (“prior skeleton”) and there is limited guidance of what this should be when little is known about this association. In this manuscript the impact of applying the CRM with different prior skeleton approaches and the 3 + 3 method are compared in terms of ability to determine the true maximum tolerated dose (MTD) and number of patients allocated to sub-optimal and toxic doses.

**Methods:**

Post-hoc dose-escalation analyses on real-life clinical trial data on an early oncology compound (AZD3514), using the 3 + 3 method and CRM using six different prior skeleton approaches.

**Results:**

All methods correctly identified the true MTD. The 3 + 3 method allocated six patients to both sub-optimal and toxic doses. All CRM approaches allocated four patients to sub-optimal doses. No patients were allocated to toxic doses from sigmoidal, two from conservative and five from other approaches.

**Conclusions:**

Prior skeletons for the CRM for phase 1 clinical trials are proposed in this manuscript and applied to a real clinical trial dataset. Highly accurate initial skeleton estimates may not be essential to determine the true MTD, and, as expected, all CRM methods out-performed the 3 + 3 method. There were differences in performance between skeletons. The choice of skeleton should depend on whether minimizing the number of patients allocated to suboptimal or toxic doses is more important.

**Trial registration:**

NCT01162395, Trial date of first registration: July 13, 2010.

**Electronic supplementary material:**

The online version of this article (doi:10.1186/s12885-016-2702-6) contains supplementary material, which is available to authorized users.

## Background

The purpose of phase 1 clinical trials is to determine the recommended dose for further clinical testing [1], whilst being efficient by minimizing the number of patients and preserving safety [2]. Trials in cancer are different to those for other indications as patients have a metastatic disease and have exhausted other treatment options [3]. Because of this, potential efficacy is also of major importance to patients, [4–6] investigators [3] and regulatory authorities [7], thus minimising the number of patients allocated to suboptimal doses is also important. Despite this, literature reviews found less than 5 % of patients in oncology trials experience a response [3, 8] and this number is decreasing [3]. The 3 + 3 method is rule based and the most common design for dose escalation studies, with over 96 % of studies using this method [1], but is not statistically efficient as it does not use all available data to recommend the next dose level to allocate [9]. This leads to more patients than necessary receiving suboptimal doses [10, 11] and limited ability to detect the MTD.

Model-based designs such as the continual reassessment method (CRM) [12] offer an alternative to rule-based designs and use Bayesian models or maximum likelihood estimation (MLE). Both rule and model-based designs aim to determine the maximum tolerated dose (MTD), the highest dose at which a pre-specified proportion of patients experience a dose-limiting toxicity (DLT). A DLT is a side effect of a treatment that is serious enough to raise concern about that dose and its definition is decided prior to dosing. Unlike rule-based designs, model-based designs use toxicity data from all dose levels, so are more statistically efficient [1]. There are around 100 publications which demonstrate the advantage of using model based methods over rule based methods in terms of efficiency and ethical considerations [13]. In particular, studies have found that, compared to the 3 + 3 method, the CRM allocates fewer patients to suboptimal [9] and harmful doses [11, 14] and identifies the true MTD a higher proportion of the time [15, 16], reducing the likelihood of making a costly and potentially unsafe decision.

Despite the benefits of model-based methods over rule-based methods, literature reviews have identified that these methods were only used in 3.3 % of phase 1 trials between 2007 and 2008 [1] and 1.6 % of trials between 1991 and 2006 [13]. Reasons for the low uptake of these models could include hesitancy to apply a complicated”black box” algorithm [17], or a lack of practical guidance for implementing these methods [2]. Model-based methods require pre-specification of the dose-toxicity model, which consists of estimates of the prior probability of experiencing a DLT for each dose (skeleton) and the prior distribution which is the underlying confidence in the prior probabilities [2]. The prior distribution has been investigated previously [18]. When there is substantial knowledge of the dose-toxicity relationship from pre-clinical or clinical studies, it can be translated into an estimate of the prior probabilities [19]. However substantial knowledge may not always be available or the translatability of the preclinical data can be in doubt. In this situation, choice of prior probabilities is a particular challenge [19, 20] and prior probabilities may not be accurate [1]. We found limited guidance on which standard prior probabilities should be used when there is limited knowledge on dose-toxicity, which is a clear area of need. It should be noted however, that Lee Cheung 2009 proposed using indifference intervals to determine prior probabilities, rather than specifying prior probabilities, an approach which deserves some consideration [2].

We sought to compare the defined MTD and number of patients allocated to sub-optimal and toxic doses obtained using the Bayesian model CRM, with different prior skeleton approaches, and the 3 + 3 method. We did so by doing a post-hoc dose-escalation analysis using real life data from the AZD3514 study, a phase 1 clinical trial in patients with metastatic castration resistant prostate cancer (CRPC) [21]. We provide a practical example of this method using our data (Appendix) and provide recommendations in the discussion to improve the uptake of these methods.

## Methods

The source dataset was a study of patients with metastatic CRPC being given AZD3514, a selective androgen receptor downregulator [21]. Patients received doses of AZD3514 monotherapy of 100 mg once daily (QD), 250 mg QD, 500 mg QD, 1000 mg QD, 1000 mg twice daily (BID) or 2000 mg BID. At the end of that study, no patients below 2000 mg BID had met the pre-determined DLT criteria. However moderate or greater nausea and vomiting were significant tolerability concerns and caused higher doses to be considered non-tolerable [22]. Therefore, moderate or greater (CTCAE grade 2+) nausea and vomiting was retrospectively defined as a DLT. The result is a relatively unique real-world dataset of dose escalations unaffected by the subsequently-lowered DLT criteria, allowing complete capture of DLTs at each dose level up to and past MTD, with dose-doubling maintained throughout.

We created an exploratory dataset with the first six patients who completed DLT assessment from each dose level between 250 mg QD and 1000 mg BID and all four patients on 2000 mg BID. The lowest dose was omitted for simplicity, especially because it was not following a dose-doubling regime. All four patients on 2000 mg BID experienced a DLT, so it was expected that data from these four patients would be sufficient for this dose level. Because nausea and vomiting were associated with increasing dose and no patients experienced a protocol defined DLT, we defined DLT as moderate/severe/very severe (CTCAE grade 2 to 4) nausea or vomiting occurring at any time during treatment. Using this dataset we will deduce the MTD, as the highest dose where the proportion of patients experiencing a DLT is below the target toxicity dose. Doses below the MTD will be considered suboptimal, and, doses above the MTD will be considered as intolerable. This method reflects how the MTD is chosen in clinical practice.

The 3 + 3 design involves allocating three patients to the initial dose level. If no patients experience a DLT, the dose level is considered safe and the next higher dose is explored. If two or more patients experience a DLT, the dose level is considered toxic and the trial can proceed to a lower dose. If one patient experiences a DLT, then three more patients are allocated to the same dose. If no further patients experience a DLT, the dose level is considered safe but if one or more further patients experience a DLT, the dose level is considered non-tolerated. The MTD is the highest dose tolerated by >4/6 of patients that received it (i.e. at least 5 of the 6 tested). For more information refer to Jaki et al. [19] who provide a schematic display of this method.

The CRM uses a Bayesian model which assumes the probability of experiencing a DLT increases with dose [19]. We need to choose the dose toxicity model, skeleton, prior distribution and target toxicity level. A dose-toxicity model should be chosen which is consistent with our a priori belief of the relationship between dose and toxicity. Examples of common dose-toxicity models include empiric [2] and logistic [18]. The prior distribution represents the initial confidence we have of the dose-toxicity relationship and many examples of these distributions are provided by Chevret [18]. The target toxicity level is the maximum proportion of patients experiencing a DLT that is acceptable given the risk benefit profile. Initial estimates of prior probabilities of DLT for each dose form the initial dose toxicity curve (skeleton). The curve is continually updated as new patient dose-toxicity information is included. If one extra patient who experienced a DLT is included in the model, the dose toxicity curve shifts upwards indicating an increase in the probability of experiencing a DLT at all doses. If one extra patient who did not experience a DLT is included in the model, the dose toxicity curve shifts downwards, indicating a decrease in the probability of experiencing a DLT at all doses. After the model is updated, the CRM will recommend that the next patient(s) are allocated the dose which is closest to the target toxicity level. If one extra patient is added, because the curve shifts are dependent on DLTs, the next recommended dose cannot increase if a DLT is experienced, and cannot decrease if a DLT is not experienced. This is demonstrated clearly in Appendix. There are various stopping rules to determine the MTD, the simplest of which is stopping after six patients have received the same dose. Goodman’s modification involved enrolling one to three patients to each cohort, starting with the lowest dose and escalating one dose each time until the first DLT is experienced [11]. After this, the CRM method is used to determine the next dose and all further doses. The CRM method with this modification is commonly known as the extended CRM [15]. This ensures some patients receive the lowest dose which preserves safety, making the initial dose independent of the prior probabilities. If the CRM is used to identify the first dose it will recommend the one with the initial prior probability closest to the target toxicity level.

We used the extended CRM to address concerns about having adequate data from lower dose levels in this scenario where 100 % dose escalations are permitted and the dose-toxicity relationship is unknown, and started at the lowest dose level because a clear safety margin to the expected MTD dose is mandated in regulatory requirements [7]. Goodman enrolled one, two and three patients to each cohort prior to the first DLT and found no difference to accuracy [11]. We elected to use two, assuming that duplicate safety data from lower dose levels would provide adequate information for escalation to proceed. Choices of model calibration for the continual reassessment method are specified in Table 1.

**Table 1:** Model calibration for the continual reassessment method

Criteria	Possible choices	Our model
Dose-toxicity model	Empiric, logistic, power	Empiric
Prior distribution	Gaussian, exponential	Gaussian
Target toxicity level	0 to 100 %	<33 %
Stopping rule	Unlimited.	A maximum of 6 patients at a single dose.
Prior skeleton	Unlimited	Conservative, aggressive, step-up, dose-linear, sigmoidal, O’Quigley
Adaption - Extended CRM	Allocate 1 to 3 patients to each cohort prior to the CRM	Allocate 2 patients to each cohort prior to the CRM

**Fig. 1:**
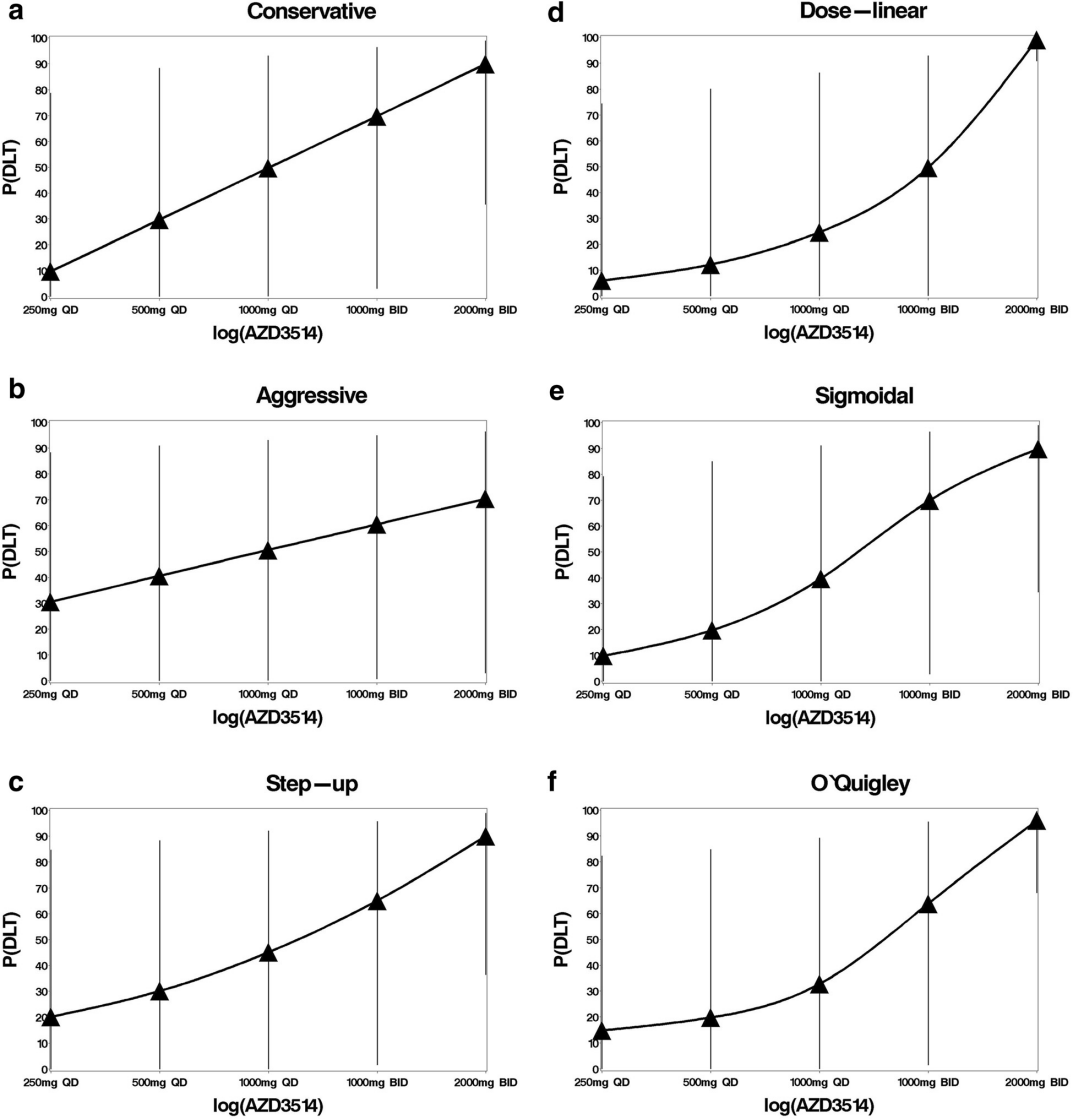
Initial dose-toxicity curves and 95 % prediction intervals from prior skeleton approaches. The predicted probabilities of experiencing a DLT and corresponding 95 % prediction intervals for each prior skeleton approach used in the extended CRM method prior to the inclusion of any dose-toxicity data

An algorithm that recommends the next dose is increased by more than one dose at a time may cause concerns about safety [11]. To explore this, we decided that when the CRM recommends a dose increase of more than one, we will continue with this recommendation. For comparison, an analysis where the dose only increases by one level will also be conducted. If the CRM recommends a dose reduction of more than one, we will apply this recommendation. We considered six prior skeleton approaches; conservative, aggressive, step-up, dose-linear, sigmoidal, and O’Quigley which are displayed in Fig. 1a. We used the empiric dose-toxicity model for all approaches as we wanted to explore a range of relationships between dose and toxicity. For the O’Quigley approach, we standardised dose values and put these into the hyperbolic distribution in order to determine the prior probabilities for this method. The dose-linear approach assumes the probability of DLT [P(DLT)] increases at the same rate as dose. The step-up, dose-linear and sigmoidal approaches were thought to roughly imitate a more typical biological relationship between dose and toxicity by assuming the difference in P(DLT) is dose-proportional and hence greater between higher dose levels than between lower dose levels, as O’Quigley et al. did [12]. However with little knowledge on dose-toxicity, it may be difficult to predict when the dose curve will rise steeply. Therefore the relationship may be correct for an infinite dose range of the drug but possibly not for the range used for the trial. The conservative and aggressive approaches were chosen as they have an even difference in P(DLT) between each dose level (log-linear to dose in this example). The conservative approach requires more knowledge that a dose is safe before moving onto the next dose than the aggressive approach. The step-up, dose-linear and sigmoidal approaches require little knowledge that a lower dose is safe to escalate, but considerable knowledge that a higher dose is safe to escalate.

Two further exploratory analyses were conducted. Firstly, we examined the effect of changing the prior P(DLT) values by adding 10 percentage points to each prior P(DLT) in each prior skeleton approach and reran the approaches. For instance, the conservative approach has P(DLT) of 10 % and 30 % for the first two doses, whereas the conservative + 10 percentage points approach has 20 % and 40 % for these doses. Increased P(DLT) should lead to slower dose-escalation as the higher doses are further away from the target toxicity level line at the start. Note for prior probabilities exceeding 100 %, the prior probability was considered to be 99 %. For instance the 2000 mg BID prior probability was 96 % for the O’Quigley approach and 99 % for the O’Quigley + 10 percentage points approach. Secondly we reproduced the extended CRM with the conservative approach but instead enrolled three patients to each dose prior to the first DLT for further comparison with the 3 + 3 method. This version of the extended CRM cannot recruit less than three patients in each cohort prior to the first DLT, so may also be appropriate in circumstances where it is desirable to have more data at lower dose levels for other dose-dependent effects, such as measure of biological activity to determine a maximum biological effective dose that may be below MTD.

### Statistical analysis

We performed extended CRM analysis with the empiric discrete dose-toxicity model, with a Gaussian prior distribution of mean 0 and variance 1.34 for each prior skeleton approach on the exploratory dataset, using the escalator package in R (https://www.r-project.org/). Target toxicity level was set to <33 % to aid comparison with the 3 + 3 method. A dose was identified as the MTD when six patients have already received this dose and the CRM recommended a 7^th^ patient receive the same dose. This aids comparison with the 3 + 3 method, because another dose may be explored after six patients, but no more than 6 patients would be in a single cohort. The CRM model choices are specified in Table 1. The 3 + 3 method was also applied to the exploratory dataset, we assumed each patient in a cohort begun their treatment simultaneously.

Each dose-escalation method will use the occurrence of DLTs at each dose as specified in the exploratory dataset (Table 2) i.e. the first patient allocated to 1000 mg QD would experience a DLT. A worked example of the extended CRM method with the conservative approach is provided in Appendix.

**Table 2:** Occurrence of DLTs

AZD3514 dose	Number eligible	Number of DLTs	Proportion that experienced a DLT	Eligible patients experiencing DLT (DLT = D, No DLT = blank)					
				1st	2nd	3rd	4th	5th	6th
250 mg QD	6	0	0 %						
500 mg QD	6	0	0 %						
1000 mg QD	6	1	16.7 %	D					
1000 mg BID	6	3	50 %		D		D	D	
2000 mg BID	4	4	100 %	D	D	D	D	-	*-*

The dose escalation approaches were compared in terms of identifying the true MTD, and the number or patients who would receive suboptimal or toxic doses.

## Results

In total, 28 patients were eligible and included in the exploratory dataset, six in each AZD3514 cohort from 250 mg QD to 1000 mg BID and four in the 2000 mg BID cohort. Of the patients receiving between 250 mg QD to 1000 mg BD AZD3514, one patient was not included because they received less than 28 days of treatment at one dose, and thirteen were not included because we had already reached the maximum quota of six patients per dose level. Eligible patients had a mean age of 69 years (range 45–79).

**Fig. 2:**
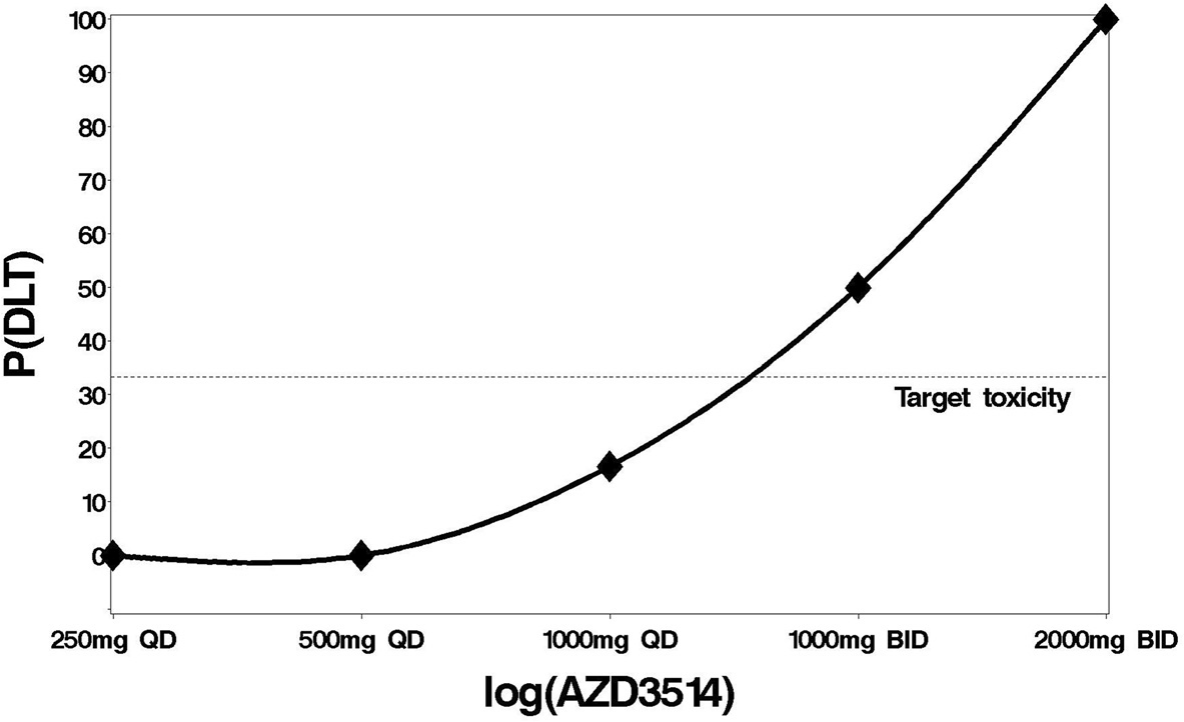
True dose toxicity curve. The observed proportion of DLTs at each dose level from the exploratory dataset

Eight of the eligible patients (29 %) experienced a DLT during treatment (Table 2, Fig. 2). These were: the first patient who received 1000 mg QD; the second, fourth and fifth patient who received 1000 mg BID; and all four patients who received 2000 mg BID. No patients experienced a DLT on 500 mg AZD3514 per day or less. This suggests there is a clear positive relationship between dose and toxicity and the dose-toxicity relationship is steeper than any of our chosen priors. We observed 1000 mg BID and 2000 mg BID are intolerable as over 33 % of patients on these doses experienced a DLT (1000 mg BID: 3/6 patients or 50 %, 2000 mg BID: 4/4 patients or 100 %). 1000 mg QD is the highest dose where the toxicity is less than 33 %, so dose escalation methods should identify this is the MTD. Therefore we considered doses below 1000 mg QD as suboptimal.

### Primary analysis

The number of patients required to identify the MTD as well as the number and proportion of patients allocated to suboptimal (250 mg QD and 500 mg QD) and intolerable (1000 mg BID and 2000 mg BID) doses for each prior skeleton approach is in Table 3. All methods correctly identified 1000 mg QD as the MTD (Table 3). However the CRM methods required 10 to 15 patients to identify the MTD, whereas the 3 + 3 method required 18 patients. The CRM methods only allocated four patients to suboptimal doses, while the 3 + 3 method allocated six patients. Four patients would have experienced DLTs if the 3 + 3 design was used, compared to one to four patients if the CRM method was used. No methods allocated any patients to the most toxic dose 2000 mg BID.

**Table 3:** Comparison of dose escalation methods

			Number of patients (Order of receiving dose – DLTs are bold)						Number of patients	
Method	Prior skeleton approach	MTD identified	250 Mg QD	500 Mg QD	1000 Mg QD	1000 Mg BID	2000 Mg BID	Total	Suboptimal (<1000 mg QD)	Intolerable (>1000 mg QD)
3 + 3	-	1000 Mg QD	3 (1, 2, 3)	3 (4, 5, 6)	6 (**7**, 8, 9, 10, 11, 12)	6 (13, **14**, 15, **16**, **17**, 18)	0	18	6	6
Extended CRM −2^a^	Conservative	1000 Mg QD	2 (1, 2)	2 (3, 4)	6 (**5**, 6, 7, 8, 9, 10)	2 (11, **12**)	0	12	4	2
	Aggressive	1000 Mg QD	2 (1, 2)	2 (3, 4)	6 (**5**, 6, 7, 10, 13, 15)	5 (8, **9**, 11, **12**, **14**)	0	15	4	5
	Step-up	1000 Mg QD	2 (1, 2)	2 (3, 4)	6 (**5**, 6, 7, 8, 9, 14)	5 (10, **11**, 12, **13**, **15**)	0	15	4	5
	Dose-linear	1000 Mg QD	2 (1, 2)	2 (3, 4)	6 (**5**, 6, 7, 8, 11, 15)	5 (9, **10**, 12, **13**, **14**)	0	15	4	5
	Sigmoidal	1000 Mg QD	2 (1, 2)	2 (3, 4)	6 (**5**, 6, 7, 8, 9, 10)	0	0	10	4	0
	O’Quigley	1000 Mg QD	2 (1, 2)	2 (3, 4)	6 (**5**, 6, 7, 8, 9, 10)	5 (11, **12**, 13, **14**, **15**)	0	15	4	5
Extended CRM −3^b^	Conservative	1000 Mg QD	3 (1, 2, 3)	3 (4, 5, 6)	6 (**7**, 8, 9, 10, 11, 14)	5 (12, **13**, 15, **16**, **17**	0	17	6	5

^a^Two patients in each cohort prior to CRM. ^b^Three patients in each cohort prior to CRM

**Fig. 3:**
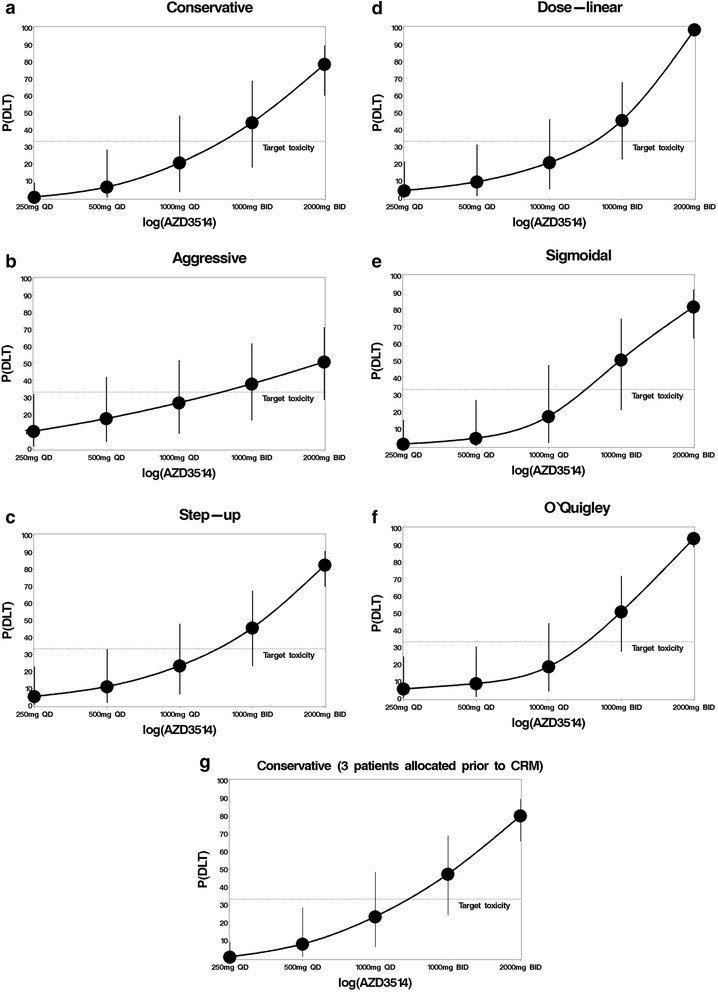
Final dose toxicity curves and 95 % prediction intervals for every CRM method. The predicted probabilities of experiencing a DLT and corresponding 95 % prediction intervals for each prior skeleton approach used in the extended CRM method after the MTD has been determined for the AZD3514 data

There were no occurrences where any CRM method recommended a dose level increase or reduction of more than one. The sigmoidal approach required the lowest number of patients (10) to correctly identify the MTD of 1000 mg QD and allocated no patients to a toxic dose, although in practice we may wish to allocate patients at the next highest dose to assess if it is tolerable. All other approaches allocated at least two patients to the non-tolerated dose of 1000 mg BID. Of these, the conservative approach required the least patients to determine the MTD (12) and allocated the lowest number of patients to toxic doses (2). The aggressive, step-up, dose-linear and O’Quigley approaches required 15 patients to determine the MTD and allocated more patients to a toxic dose (5). Notably these approaches allocated a fifth patient to 1000 mg BID despite two out of four patients at this dose already experiencing a DLT. One DLT would have been experienced if the sigmoidal approach was used to escalate dose, two if the conservative approach was used and four if any other prior skeleton approach was used. There is considerably more confidence in estimates of the P(DLT) at each dose in the final dose-toxicity curves (Fig. 3a to f) than the prior dose-toxicity curves (Fig. 1a to f), as expected. The final dose-toxicity curves have similar distributions between the lowest and second highest dose and prediction intervals to each other and are somewhat similar to the true distribution (Fig. 2). The P(DLT) varies considerably for the highest dose, which is probably because no patients were tested at this level. There were two notable differences, the P(DLT) for 1000 mg BID is noticeably higher in the sigmoidal and dose-linear approaches and the aggressive approach did not achieve a strong curve like the other approaches. The credible intervals for the P(DLT) of the 1000 mg BID was widest in the conservative and sigmoidal approaches, which is probably because less patients were tested at this dose then in other approaches.

### Exploratory analysis

Adding 10 percentage points to all priors in the sigmoidal, step-up and aggressive approaches made no difference to their dose allocation and all approaches still correctly identified the correct MTD, interestingly with the same number of patients or less (Additional file 2). The dose-linear approach required one less patient to achieve the MTD, which decreased the frequency of patients allocated to toxic dose by one and in doing so reduced the number of DLTs experienced from four to three. The conservative approach also required one less patient to achieve the MTD, one additional patient was allocated to a suboptimal dose, and two less patients were allocated to toxic doses which reduced the number of DLTs experienced from two to one. The O’Quigley approach required five less patients to identify the MTD, and the number of patients allocated to 1000 mg BID (toxic dose) reduced from five to zero, which reduced the number of DLTs experienced from four to one. Adding 10 percentage points to each prior made little difference to the final P(DLT) for the lowest three doses and dose-toxicity distribution of any prior skeleton approach (Additional file 1).

When the conservative approach was rerun with three patients allocated to each dose prior to the first DLT occurring, the approach required five additional patients to determine the DLT (Table 3). Two additional patients received suboptimal doses and three additional patients received toxic doses of 1000 mg QD which would result in two more DLTs. This approach allowed no reductions in suboptimal doses compared to the 3 + 3 method but did allocate one less patient to a toxic dose, which also resulted in one less patient overall. The final dose toxicity curve (Fig. 3g) was similar to that of the conservative approach with two patients at each dose (Fig. 3a).

## Discussion

This post-hoc analysis on clinical dose-escalation data compared the CRM method with various prior skeleton approaches and the 3 + 3 method. The results provide further evidence the CRM method is more efficient and may preserve safety compared to the 3 + 3 method as every prior skeleton approach required less patients to identify the MTD, and allocated less patients to suboptimal and toxic doses. It is notable that the CRM outperformed the 3 + 3 even though the true dose-toxicity curve was steeper than any of our chosen prior skeleton approaches. We found the underlying model of the dose-toxicity relationship influences the number of patients allocated to toxic doses, but in all cases the correct optimal dose was chosen.

To our knowledge this is the first study to compare prior skeleton approaches in the CRM method. O’Quigley & Chevret also found that even if prior probabilities are underestimated or overestimated the performance of the CRM will be at least as good as standard methods [16]. Lee & Cheung observed that most studies use the O’Quigley et al. [12] prior skeleton approach without providing justification [2]. Many dose escalation studies that we identified did not display the prior probabilities they used or justify how they obtained them.

For our data, the conservative prior skeleton approach was more successful then the step-up and dose-linear approaches as it allocated less patients to toxic doses despite the original dose-toxicity curves of these approaches being closer to the true relationship. This may suggest the overall spacing between prior probabilities is a key factor of the dose-toxicity relationship in the original prior combination, and the spacing may be more important than the overall shape of the curve. Another plausible dose-toxicity relationship is the one used in the sigmoidal approach, but no patients were allocated to 1000 mg BID despite only one out of six patients at the dose below experiencing a DLT. If the spacing between 1000 mg QD and 1000 mg BID was closer, then some patients may have been allocated to the next highest dose which highlights the strong barrier to dose escalation that the steep part of the curve presents. One concern was that the O’Quigley, aggressive, step-up and dose-linear approaches allocated a further patient to 1000 mg BID despite two out of four patients on this dose experiencing a DLT. This could be caused by insufficient spacing between the P(DLT) for 1000 mg QD and 1000 mg BID (10 % aggressive, 20 % step-up, 25 % dose-linear and 29 % O’Quigley approaches) or prior probabilities of the toxicity of the 1000 mg BID dose not high enough (50 % dose-linear, 60 % aggressive, 64 % O’Quigley and 65 % step-up approaches). Notably in the conservative and O’Quigley approaches when the prior probability of 1000 mg BID was 64 % and 70 % respectively, some patients received this dose, when it was 74 % and 80 % respectively (conservative + 10 percentage points and O’Quigley + 10 percentage points approach) no patients received this dose. The highest prior probability to receive any dose was 75 % and was the 1000 mg BID dose from the step-up + 10 percentage points approach.

To escalate faster and reduce the number of patients on suboptimal doses, we could lower the P(DLT) prior probabilities but this would put more patients at risk of a DLT, which causes a suboptimal/toxic dose ethical dilemma. Therefore choice of prior skeleton approach for studies should partially depend on which of minimising suboptimal or minimising toxic doses is more important. Daugherty et al. reported a cancer trial where patients got to select their own dose and found patients would chose the highest dose even with knowledge of the increased toxicity risk and patients thought more about possible benefits than side effects when choosing their dose [23].

Clinical opinion should also be used in decisions to recommend the next dose to improve the flexibility of choice. We identified two situations where investigators may have wished to override the CRM decision. Firstly, where one dose is considered safe (i.e. target toxicity not exceeded), but at the dose level above, either no patients (sigmoidal approach) or a small number of patients (conservative approach) have been tested, the investigators may wish to test more patients at the higher dose, thus overriding the CRM decision. Secondly, other CRM prior skeleton approaches allocated a patient to 1000 mg BID despite two of four patients who had already received this dose experiencing a DLT, investigators may wish to stop this extra patient receiving this dose. Including an additional modification to the CRM method such as escalation with overdose control (EWOC) may also prevent too many patients receiving a toxic dose [24].

We chose a stopping rule to be a maximum of 6 patients treated at a single dose and identified a dose as the MTD when the CRM recommended a 7th patient to receive the same dose. This enabled a direct comparison of CRM with the 3 + 3 design. To increase confidence in dose-toxicity relationship and the prediction of MTD, it is possible to allocate additional patients to dose levels. Clinical opinion is also important in the 3 + 3 method. For the 1000 mg BID dose, one out of the first three patients experienced a DLT. For the 4^th^, 5^th^ and 6^th^ patients allocated to this dose, a clinician may have suggested allowing time between these patients receiving their first doses, which may prevent more than one DLT occurring in these patients.

Since this study was designed to compare effects of different models on MTD assessment, the scenario chosen was one where toxicity is assumed to be the primary determinant of the recommended phase II dose. Pharmacodynamic response could be modelled in a similar way and determination of a maximum biological effective dose would be expected to raise similar issues of rule-based systems versus statistically efficient model-based systems or Bayesian approaches.

### Strengths + limitations

This study has several strengths. It is a post-hoc analysis on a real phase 1 clinical trial, there were at least six eligible patients at four doses, the probability of DLT increased markedly with increased dose and information on patient characteristics was available, and we considered several skeleton scenarios that covered a range of prior beliefs of toxicity. A limitation of this study is that we did not consider time between each patient being allocated a dose. In practice, this process could be sped up by allocating doses to two patients at a time.

### Implications

This research has implications for future phase I trials. Further support is provided for using the CRM instead of standard methods. The importance of selecting an appropriate prior dose-toxicity model has been shown. Specifying a wide prediction interval for each prior probability allows the model to be influenced by the data so highly accurate estimates of each prior probability may not be essential to determine the true MTD. Several prior dose-toxicity models have been proposed, and compared, and recommendation made for their use in future trials.

## Conclusions

The CRM model is more efficient and may expose less patients to toxic doses compared to the 3 + 3 method, even when the optimal dose-toxicity curve is unknown. Choice of the prior skeleton approach and initial estimates should depend on whether minimizing the number of patients allocated to suboptimal or toxic doses is more important. Highly accurate initial estimates may not be essential to determine the true MTD. This manuscript describes prior dose-toxicity models that could be used when limited dose-toxicity relationship data is available and raises the importance of further exploration into this. It also reiterates the importance of combining the CRM recommendations with clinical opinion for decisions to escalate/de-escalate dose. We advise authors who are using CRM methods to make available their initial priors and final dose-toxicity graphs so optimal generic graphs can be derived and to support the uptake of these methods.

## Figures and Tables

**Fig. 4:**
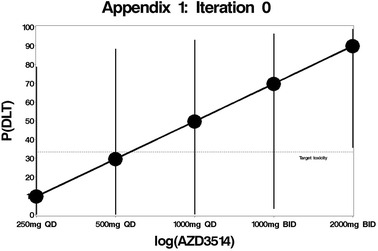
The predicted probabilities of experiencing a DLT and corresponding 95 % prediction intervals for the conservative prior skeleton approach in the CRM method prior to the inclusion of any dose-toxicity data

**Fig. 5:**
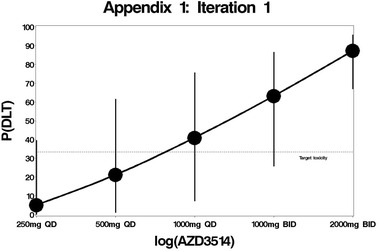
The predicted probabilities of experiencing a DLT and corresponding 95 % prediction intervals for the conservative prior skeleton approach in the extended CRM method after the inclusion of the first five AZD3514 patients

**Fig. 6:**
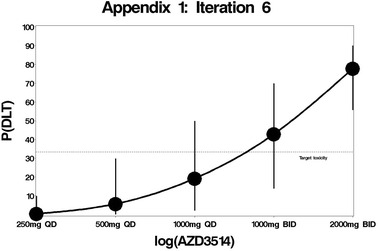
The predicted probabilities of experiencing a DLT and corresponding 95 % prediction intervals for the conservative prior skeleton approach in the extended CRM method after the inclusion of the first ten AZD3514 patients

**Fig. 7:**
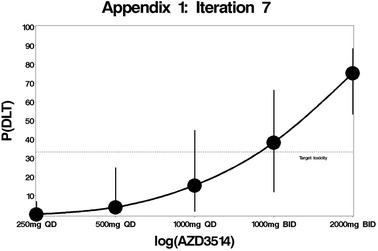
The predicted probabilities of experiencing a DLT and corresponding 95 % prediction intervals for the conservative prior skeleton approach in the extended CRM method after the inclusion of the first eleven AZD3514 patients

**Fig. 8:**
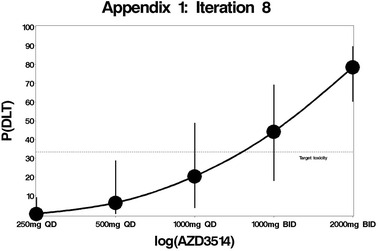
The predicted probabilities of experiencing a DLT and corresponding 95 % prediction intervals for the conservative prior skeleton approach in the extended CRM method after the MTD has been determined for the AZD3514 data

## Appendix

### A worked example of the extended CRM with conservative prior skeleton approach

The order of patients who experienced DLTs is in Table 2. The original dose-toxicity curve of the conservative approach and corresponding 95 % prediction interval is below.

Two patients receive 250 mg QD. Neither experienced a DLT so can escalate to next dose.

Two patients receive 500 mg QD. Neither experienced a DLT so can escalate to next dose.

The first patient who receives 1000 mg QD experiences a DLT.

The dose toxicity curve is updated with the above toxicity information as displayed below.

From now on, the CRM will be used to determine the next dose.

### Iteration 1

The CRM recommends the next patient receives dose 3 (1000 mg QD) as this dose has the closest P(DLT) to the target toxicity level (33 %).

The next patient receives 1000 mg QD, does not experience a DLT and the CRM method recommends the next dose is dose 3 (1000 mg QD). This happens four more times.

### Iteration 6

The CRM recommends the next patient receives dose 4 (1000 mg BID). One patient receives this dose, does not experience a DLT and the CRM method recommends the next dose is dose 4 (1000 mg QD).

### Iteration 7

The CRM recommends the next patient receives dose 4 (1000 mg BID). One patient receives this dose, experiences a DLT and the CRM method recommends the next dose is dose 3 (1000 mg QD).

### Iteration 8

Six patients have already received 1000 mg QD and this is the next recommended dose which meets our stopping criteria. We stop the trial and 1000 mg QD is identified as the MTD.

## Additional files

Additional file 1: Comparison of dose escalation methods for + 10 percentage points approaches. (DOCX 32 kb) Additional file 2: Final dose toxicity curves and 95 % prediction intervals for every CRM method + 10 percentage points. Legend: The predicted probabilities of experiencing a DLT and corresponding 95 % prediction intervals for each prior skeleton + 10 % approach used in the extended CRM method after the MTD has been determined for the AZD3514 data. (ZIP 678 kb)
