# Supplementary material for: Continual reassessment method for dose escalation clinical trials in oncology: a comparison of prior skeleton approaches using AZD3514 data
Source: BMC Cancer. 2016 Aug 31;16(1):703. doi: 10.1186/s12885-016-2702-6 (PMC5007718; doi:10.1186/s12885-016-2702-6)
Supplement: Additional file 1: — Comparison of dose escalation methods for + 10 percentage points approaches. (DOCX 32 kb) [file 12885_2016_2702_MOESM1_ESM.docx]

# Tables

# Additional file

Additional file 1: *Comparison of dose escalation methods for + 10 percentage points approaches*

|  |  |  | **Number of patients (Order of receiving dose)** | | | | | | **Number of patients** | |
| --- | --- | --- | --- | --- | --- | --- | --- | --- | --- | --- |
| **Method** | **Prior combination** | **MTD identified** | **250 Mg**  **QD** | **500 Mg**  **QD** | **1000 Mg**  **QD** | **1000 Mg**  **BID** | **2000 Mg**  **BID** | **Total** | **Suboptimal**  **(<1000mg QD)** | **Intolerable**  **(>1000mg QD)** |
| Extended CRM -2* | **Conservative** | 1000 Mg QD | 2  (1, 2) | 3  (3, 4, 6) | 6  (**5**, 7, 8,  9, 10, 11) | 0 | 0 | 11 | 5 | 0 |
|  | **Aggressive** | 1000 Mg QD | 2  (1, 2) | 2  (3, 4) | 6  (**5**, 6, 7  8, 11, 15) | 5  (9, **10**, 12, **13**, **14**) | 0 | 15 | 4 | 5 |
|  | **Step-up** | 1000 Mg QD | 2  (1, 2) | 2  (3, 4) | 6  (**5**, 6, 7,  8, 9, 10) | 4  (11, **12**, 13  **14**) | 0 | 14 | 4 | 4 |
|  | **Dose-linear** | 1000 Mg QD | 2  (1, 2) | 2  (3, 4) | 6  (**5**, 6, 7  8, 9, 15) | 5  (10, **11**, 12, **13**, **14**) | 0 | 15 | 4 | 5 |
|  | **Sigmoidal** | 1000 Mg QD | 2  (1, 2) | 2  (3, 4) | 6  (**5**, 6, 7,  8, 9, 10) | 0 | 0 | 10 | 4 | 0 |
|  | **O’Quigley** | 1000 Mg QD | 2  (1, 2) | 2  (3, 4) | 6  (**5**, 6, 7,  8, 9, 10) | 0 | 0 | 10 | 4 | 0 |

*Two patients in each cohort prior to CRM.
