# Supplementary figures and images for: Continual reassessment method for dose escalation clinical trials in oncology: a comparison of prior skeleton approaches using AZD3514 data
Source: BMC Cancer. 2016 Aug 31;16(1):703. doi: 10.1186/s12885-016-2702-6 (PMC5007718; doi:10.1186/s12885-016-2702-6)

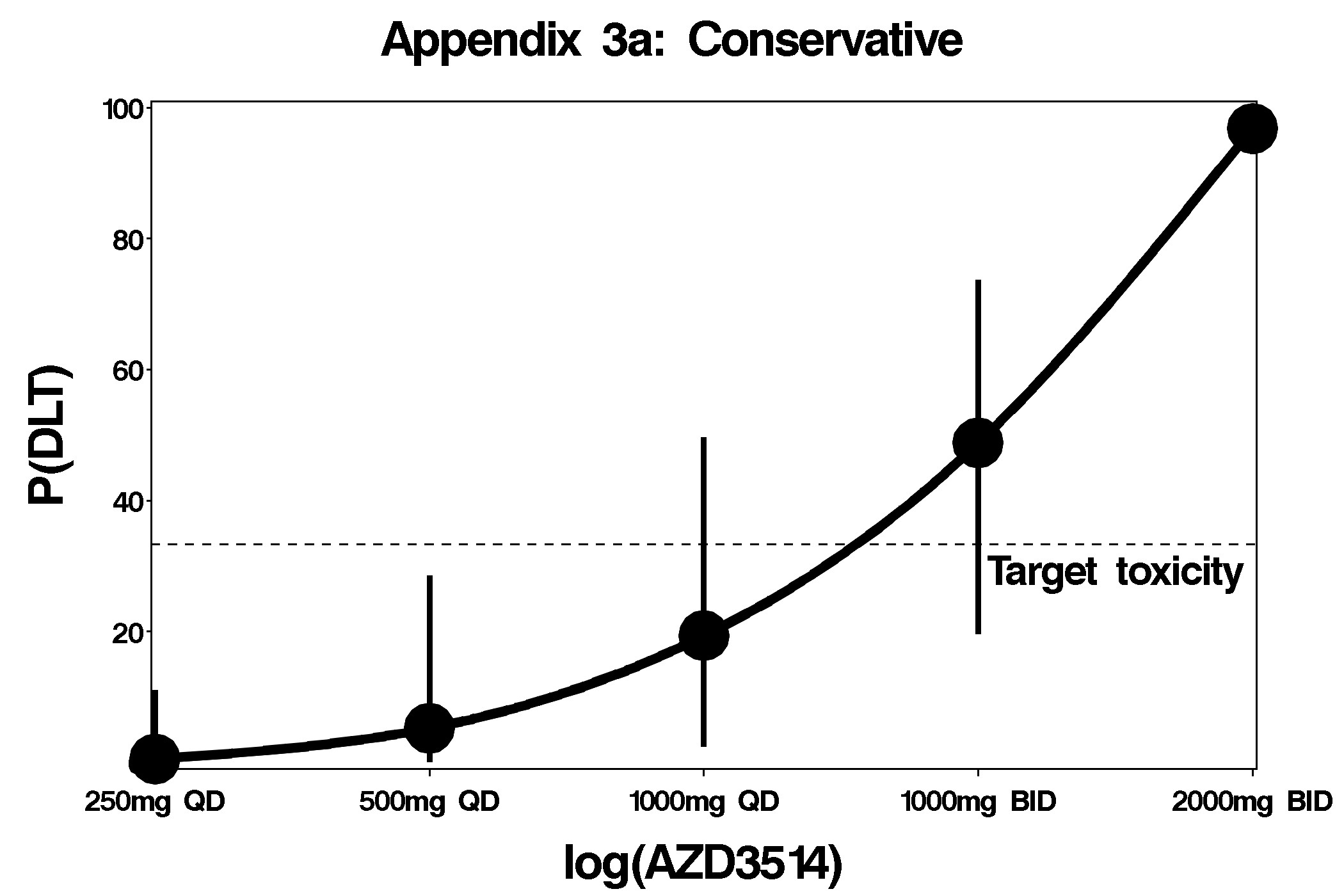

Supplement: Additional file 2: — Final dose toxicity curves and 95 % prediction intervals for every CRM method + 10 percentage points. Legend: The predicted probabilities of experiencing a DLT and corresponding 95 % prediction intervals for each prior skeleton + 10 % approach used in the extended CRM method after the MTD has been determined for the AZD3514 data. (ZIP 678 kb) [file 12885_2016_2702_MOESM2_ESM.zip › Appendix 3a Conservative posterior.jpg]

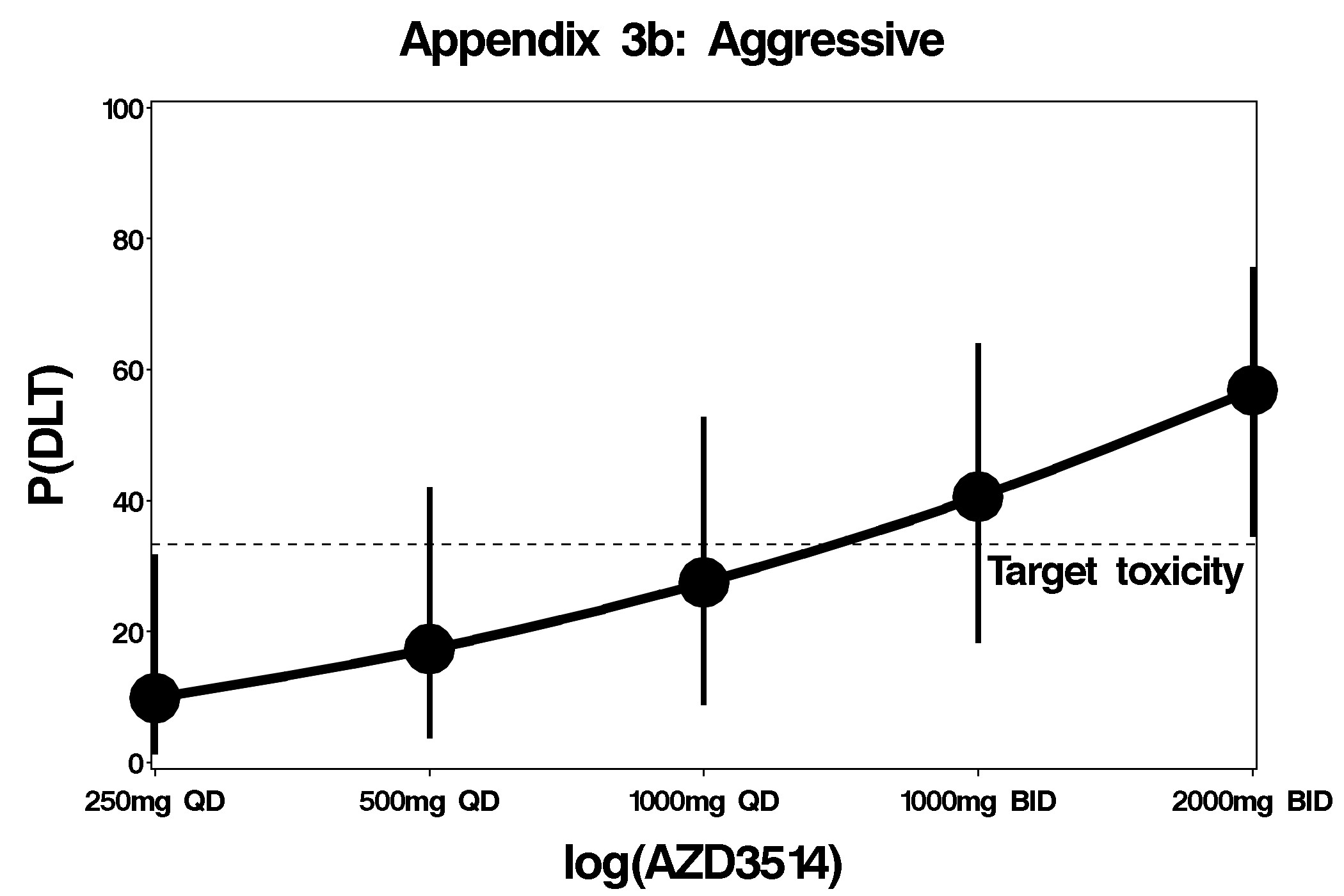

Supplement: Additional file 2: — Final dose toxicity curves and 95 % prediction intervals for every CRM method + 10 percentage points. Legend: The predicted probabilities of experiencing a DLT and corresponding 95 % prediction intervals for each prior skeleton + 10 % approach used in the extended CRM method after the MTD has been determined for the AZD3514 data. (ZIP 678 kb) [file 12885_2016_2702_MOESM2_ESM.zip › Appendix 3b Aggressive posterior.jpg]

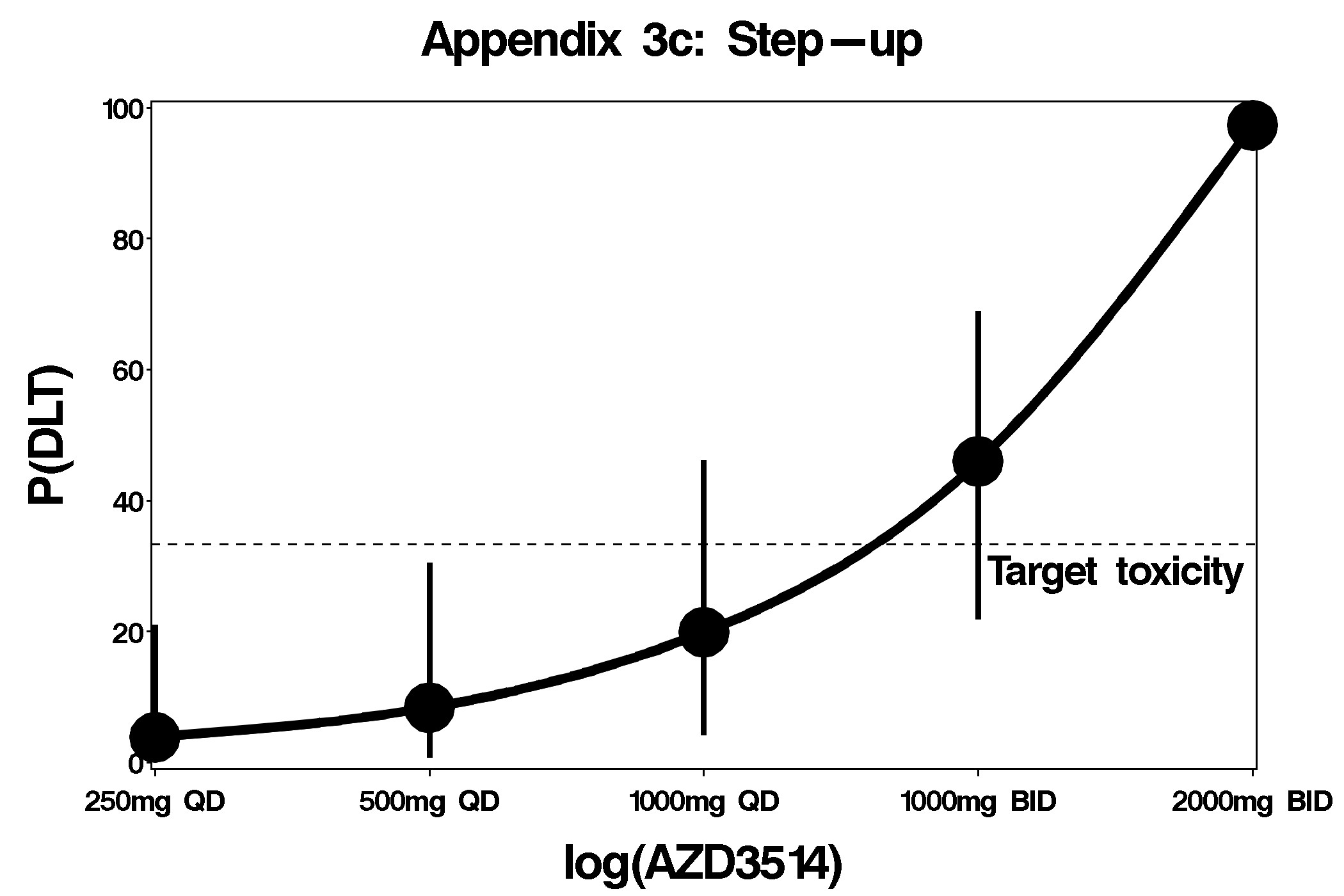

Supplement: Additional file 2: — Final dose toxicity curves and 95 % prediction intervals for every CRM method + 10 percentage points. Legend: The predicted probabilities of experiencing a DLT and corresponding 95 % prediction intervals for each prior skeleton + 10 % approach used in the extended CRM method after the MTD has been determined for the AZD3514 data. (ZIP 678 kb) [file 12885_2016_2702_MOESM2_ESM.zip › Appendix 3c Step-up posterior.jpg]

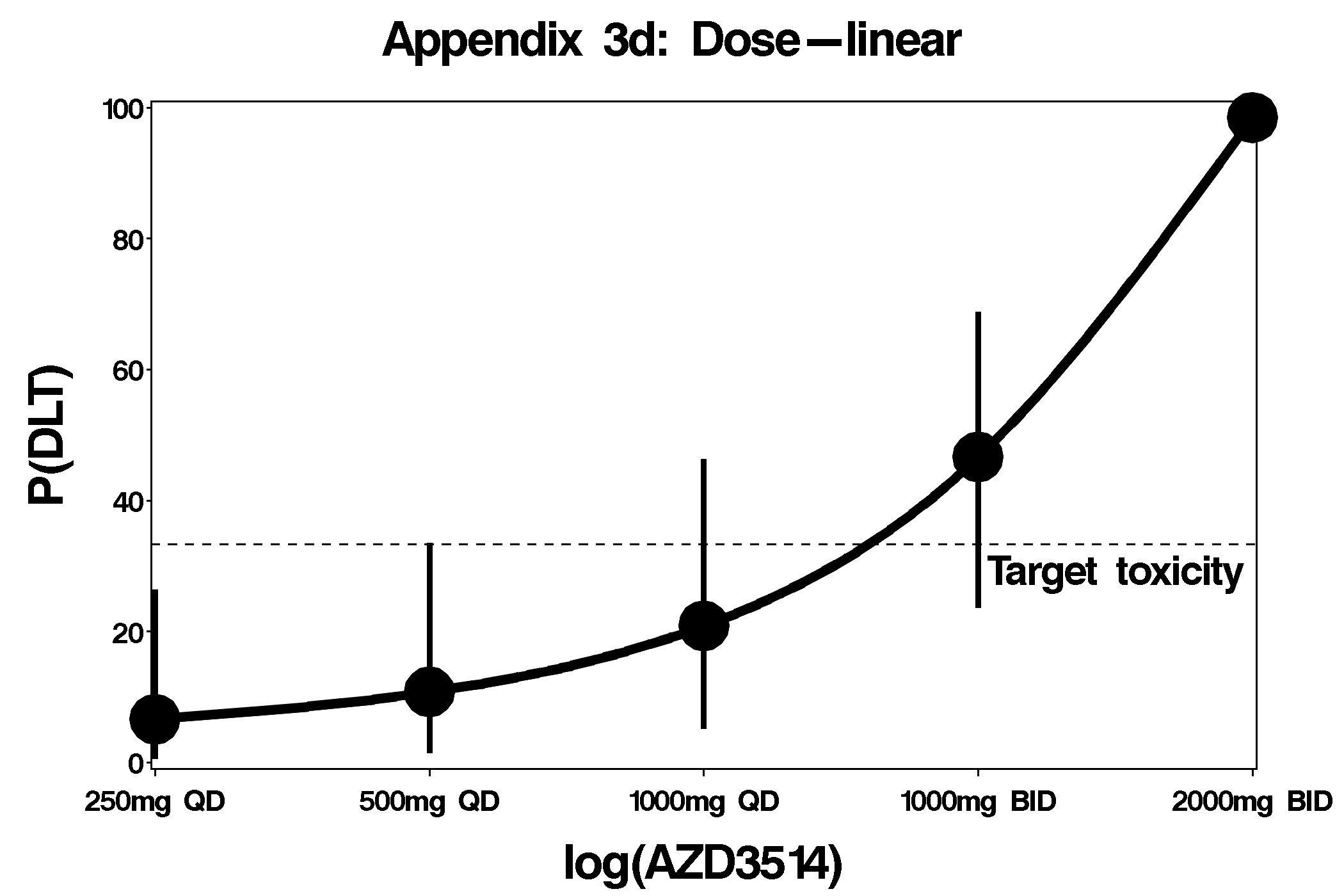

Supplement: Additional file 2: — Final dose toxicity curves and 95 % prediction intervals for every CRM method + 10 percentage points. Legend: The predicted probabilities of experiencing a DLT and corresponding 95 % prediction intervals for each prior skeleton + 10 % approach used in the extended CRM method after the MTD has been determined for the AZD3514 data. (ZIP 678 kb) [file 12885_2016_2702_MOESM2_ESM.zip › Appendix 3d Dose-linear posterior.jpg]

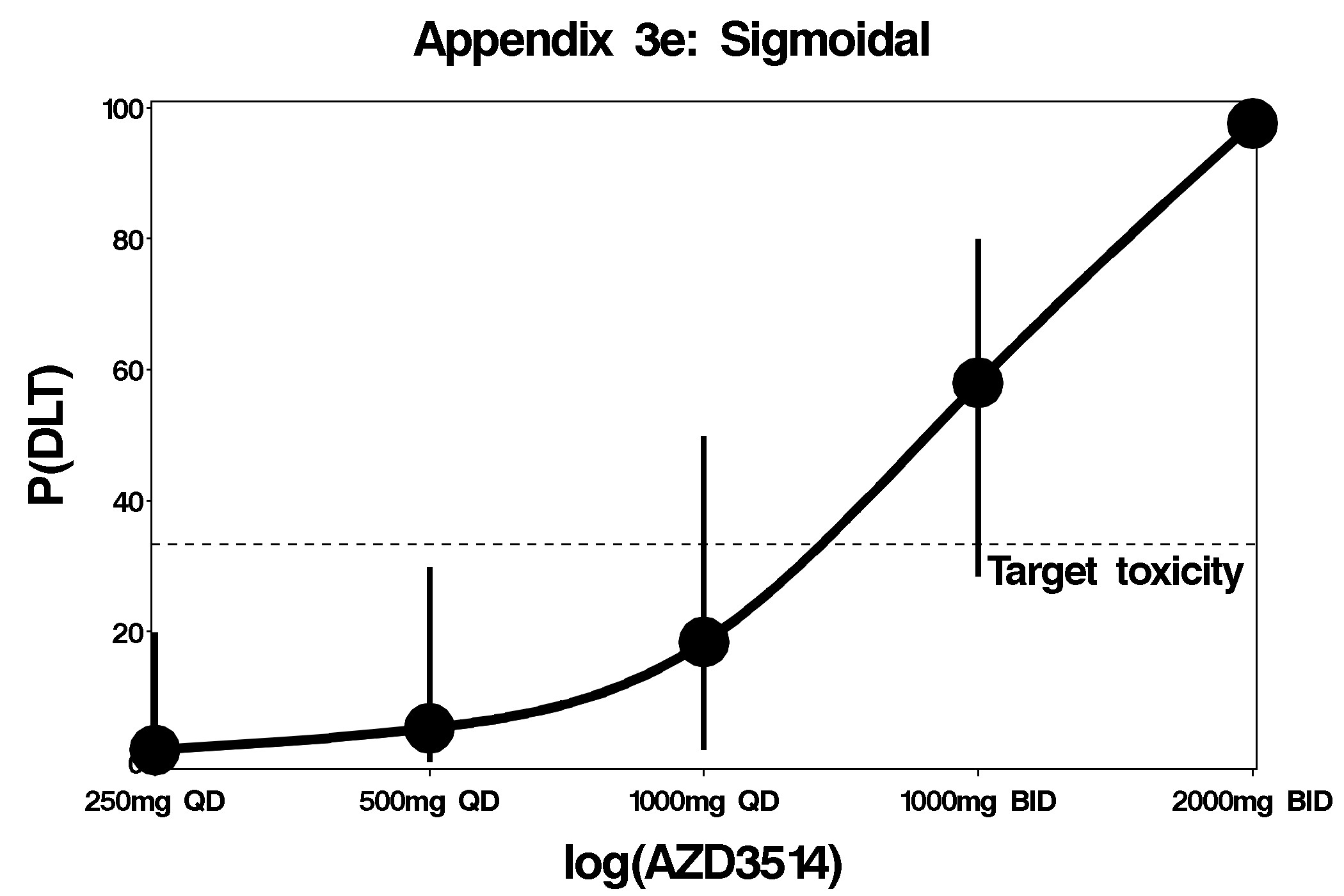

Supplement: Additional file 2: — Final dose toxicity curves and 95 % prediction intervals for every CRM method + 10 percentage points. Legend: The predicted probabilities of experiencing a DLT and corresponding 95 % prediction intervals for each prior skeleton + 10 % approach used in the extended CRM method after the MTD has been determined for the AZD3514 data. (ZIP 678 kb) [file 12885_2016_2702_MOESM2_ESM.zip › Appendix 3e Sigmoidal posterior.jpg]

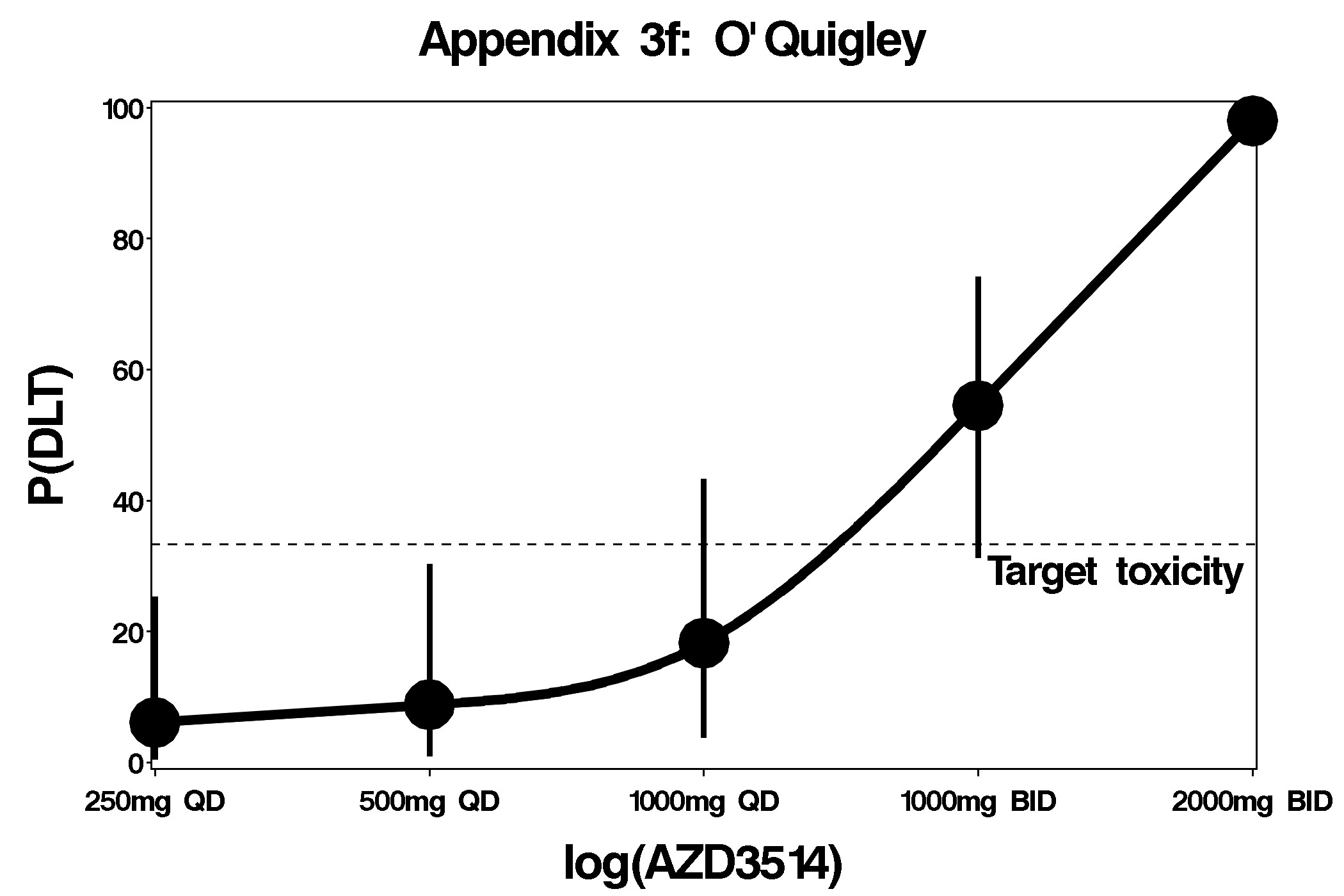

Supplement: Additional file 2: — Final dose toxicity curves and 95 % prediction intervals for every CRM method + 10 percentage points. Legend: The predicted probabilities of experiencing a DLT and corresponding 95 % prediction intervals for each prior skeleton + 10 % approach used in the extended CRM method after the MTD has been determined for the AZD3514 data. (ZIP 678 kb) [file 12885_2016_2702_MOESM2_ESM.zip › Appendix 3f O'Quigley posterior.jpg]
